# A Special Issue Introduction: Educational Applications of Cognitive Psychology

**DOI:** 10.3390/bs16050713

**Published:** 2026-05-06

**Authors:** Jeri L. Little, Elizabeth Ligon Bjork

**Affiliations:** 1 Department of Psychology, California State University, East Bay, Hayward, CA 94542, USA; 2 Department of Psychology, University of California, Los Angeles, Los Angeles, CA 90095, USA; elbjork@psych.ucla.edu

## 1. Introduction

How to optimize knowledge retention and transfer is a fundamental question for students and educators. Recently, the “science of learning” has received widespread interest in popular books like *Make It Stick* (P. C. Brown et al., 2014) and *How We Learn* (Carey, 2015). By examining how people process, store, and retrieve information, cognitive psychologists have identified key mechanisms and processes that underlie effective learning and have developed practical strategies for enhancing learning outcomes (e.g., Dunlosky et al., 2013; Mayer, 2008; Roediger & Pyc, 2012). Additionally, cognitive psychologists are interested in learners’ beliefs (i.e., metacognition) about how learning occurs and, particularly, under what circumstances learners’ judgments of their own learning misalign with their actual learning outcomes. Finally, cognitive psychologists are increasingly interested in individual differences that can influence the effectiveness of these strategies.

Most of the articles within this Special Issue align with the central themes in the science of learning, including desirable difficulties, metacognition, and individual differences. At the same time, several contributions extended beyond these central themes, reflecting the breadth of contemporary research at the intersection of cognitive psychology and education. Such contributions highlight emerging directions for the science of learning.

In this Introduction, we focus on some of the major research findings on how to optimize retention and transfer and what learners know about these processes, highlighting the publications included in this Special Issue. We then offer practical recommendations and provide avenues for future research.

## 2. Improving Knowledge Retention with “Desirable Difficulties”

One powerful idea in the science of learning is that some strategies that feel difficult to the learner are actually desirable for long-term retention (R. A. Bjork, 1994; R. A. Bjork & Bjork, 2011, 2020; A. Nelson & Eliasz, 2023). Such “desirable difficulties” include testing oneself rather than rereading (Roediger & Karpicke, 2006; Roediger & Butler, 2011), spacing out study sessions rather than massing them (Cepeda et al., 2006, 2008; Dempster, 1989), and interleaving the presentation or study of items from different categories rather than blocking items by category (Brunmair & Richter, 2019; Firth et al., 2021; Kornell & Bjork, 2008a; Pan, 2015). The desirable difficulty framework builds upon Craik and Lockhart’s (1972) levels of processing results: deeper processing of to-be-learned material increases the likelihood that it will be remembered later. To illustrate why testing is beneficial, answering a question about to-be-learned information often involves deeper processing than simply looking at it again. To illustrate why spacing is beneficial, when learners are exposed to an item multiple times, they will likely think about that item more deeply on successive presentations if time has passed since the previous presentation than if it has not, most likely because they should recognize that their memory of the item has dissipated during that interval. Finally, to illustrate why interleaving is beneficial, if learning items from different categories, learners will tend to process the second item from the category more deeply if it follows an item from a different category than if it follows an item from the same category. This increased learning is likely occurring because seeing items from different categories induces a consideration of what is making these items belong to different categories, while also considering the features necessary for membership in a given category. Considering these conditions of learning together, in the cases of testing, spacing, and interleaving, learners may process items more deeply because those items are less accessible in memory. That lower accessibility of the items when eventually retrieved in a test or seen again in a spaced or interleaved manner—while perhaps making those conditions of learning seem more difficult to the learner—is actually making that learning experience more potent and long-lasting (R. A. Bjork & Bjork, 1992; but see Kornell, 2025).^1^

The testing effect and spacing effect are generally robust across participants and contexts (e.g., Carpenter et al., 2022; Dunlosky et al., 2013) and are often used together to promote learning (Carpenter & DeLosh, 2005; Hser & Wickens, 1989; Karpicke & Bauernschmidt, 2011; Karpicke & Roediger, 2007; Landauer & Bjork, 1978; Soderstrom et al., 2016). The popular use of flashcards, for example, makes use of both testing and spacing (Kornell, 2009; Kornell & Bjork, 2008b). In the cases of both testing and spacing, the learning advantage of the “difficult” condition (testing and spacing vs. restudy and massing, respectively) is often influenced by the retention interval (i.e., the time period between the learning task and a final criterion test). Although it is sometimes the case that on an immediate test, the “easy” condition outperforms the “difficult” condition, the benefit of the “difficult” condition is typically present at a delay (e.g., Roediger & Karpicke, 2006). Critically, strong performance for the items presented in the “easy” condition on immediate tests (or simply knowing that one could regurgitate knowledge immediately after learning) is likely a major reason why learners typically overestimate the efficacy of these “undesirable” conditions for long-term learning (R. A. Bjork & Bjork, 2011). Additionally, learners tend to underestimate the amount of forgetting that occurs over time (Koriat et al., 2004), and items that are just above the threshold for successful retrieval will be easily forgotten (i.e., unable to be retrieved, Kornell & Bjork, 2025).

Although, in general, the testing and spacing effects are robust over many populations and contexts, the optimal difficulty for maximizing learning can also depend on other factors, and knowing these factors can increase the probability of learning success (c.f., Jenkins, 1979; McDaniel & Einstein, 1989). Jenkins (1979), for example, has proposed that in addition to the orienting task (e.g., desirable difficulty), three categories of factors can interact to influence memory: (a) learner characteristics, (b) materials, and (c) features of the criterial test. For learner characteristics, the optimal difficulty could depend on the background of the learner because a “desirable” level of difficulty for one person may be too difficult for another. For example, in the case of learning motor skills and sports, blocking is sometimes better than interleaving for novices, while interleaving is often better than blocking for the more advanced learner (Wulf & Shea, 2002). Other features of the task can influence whether a difficulty is desirable. In this Special Issue, Healy et al. (2025) used a variety of factors that introduce difficulties in language learning to examine which difficulties best help learning. In one experiment, they had participants study English-French translations in small or large blocks, with the idea that larger blocks may offer a desirable difficulty because of the increased spacing. Mostly consistent with those predictions, they found that although smaller blocks promoted better performance faster than did larger blocks during acquisition, that advantage did not persist on the delayed test. Similarly, although practicing pairs in the French–to-English direction sped correct performance as compared to practicing in the English-to-French direction, that advantage of French-to-English practice did not persist on a delayed test. Although Healy et al. did not find an advantage of the “difficult” condition at the delayed test, they did find an interaction between difficulty and retention interval such that the advantage of the “easy” condition was eliminated over time.

### 2.1. Tests Serve as Tools for Learning, Not Just Assessment

As previously mentioned, the testing effect (or retrieval practice effect, as it is often called) is the finding that performance on a later test is improved by using retrieval or other testing procedures during practice as compared to re-exposure or restudy of the to-be-learned content (Roediger & Karpicke, 2006). This empirical advantage of retrieval has existed in the psychological literature since the early 20th century (Gates, 1917; Spitzer, 1939). Since then, it has been shown with a wide variety of materials, contexts, and age groups (Adesope et al., 2017; Agarwal et al., 2021; Pan & Rickard, 2018; Roediger & Butler, 2011; Rowland, 2014; Yang et al., 2021) and thus has clear implications for education. An enlightening review of the use of retrieval practice in health professions by Serra et al. (2025) appears in this Special Issue.

Although research has clearly shown that, in general, testing tends to be advantageous for learning, research has also shown that a variety of factors can influence the size of that benefit. The format of the test question (e.g., short-answer, multiple-choice) is one such factor. Early research examining format indicated that cued-recall tests (e.g., short-answer) tended to result in larger learning advantages than did recognition tests and attributed that benefit to the retrieval processes afforded by cued-recall testing (Foos & Fisher, 1988). Because it was believed that performance on multiple-choice tests relies on recognition, whereas performance on cued-recall tests requires retrieval, a prevailing idea was that cued-recall tests would usually be better than multiple-choice tests for producing learning. We (E. L. Bjork et al., 2014; Little, 2018; Little & Bjork, 2015; Little et al., 2012, 2019) have argued that although multiple-choice tests can be written to rely on recognition (and less depth of processing), they can also be written to induce recall—namely, when the incorrect alternatives are competitive (plausible) answer choices. For example, Little and Bjork (2015) had participants read passages and then answer multiple-choice questions about the information in those passages that either had competitive incorrect alternatives or non-competitive incorrect alternatives. Then, on a different test administered after a short delay, participants were better able to answer questions about incorrect alternatives when they had previously been competitive alternatives than when they had been non-competitive alternatives. Additionally, Little et al. (2012) showed that multiple-choice tests could be as or more effective than cued-recall tests for retention–both of previously tested and related content–even when feedback was provided on the initial test.

Using Little and Bjork’s (2015) materials, Sparck et al. (2016) had participants give confidence-weighted responses for the alternatives (i.e., if participants were unsure of the correct answer, they could indicate which answers they were leaning toward and whether they were leaning closer to one answer or another) and found this procedure to lead to an even larger advantage for the non-tested related information than did answering standard multiple-choice questions. This finding is consistent with the idea that confidence-weighted judgments promote even deeper processing than do simple multiple-choice questions with competitive alternatives. In a similar vein, in this Special Issue, Clark et al. (2025) manipulated whether or not participants needed to justify their answer to multiple-choice questions as a means to examine the role of answer justification in improving the benefit of the testing effect with multiple-choice questions. They found that justifying answers increased the learning benefits of answering multiple-choice questions (see also Little et al., 2019).

Usually, the testing effect is examined in situations in which retrieval is overt (i.e., participants need to report what they retrieved in the form of answers on an initial test). Sometimes, however, retrieval is not or cannot be overt, and instead, students only retrieve information in their minds (Witherby et al., 2025b). Some past research has shown that whether retrieval is overt or covert has little effect on learning (Higham et al., 2023; Putnam & Roediger, 2013; Smith et al., 2013), but other research has shown overt retrieval to be more effective than covert retrieval (Abel & Roediger, 2018; Jönsson et al., 2014; Kubik et al., 2020; Sumeracki & Castillo, 2022; Tauber et al., 2018). While the above research was mostly conducted in the laboratory, Witherby et al. (2025b) examined the relative benefits of overt versus covert retrieval in 8th-grade science classes. They obtained preliminary evidence that overt retrieval might be more effective than covert retrieval in an educational setting, but cautioned that this result may have been found because learners failed to engage in retrieval processes when—in the covert condition—they did not need to show evidence of having engaged in such efforts.

Testing can also be beneficial when it occurs before learning, as in the form of pretests (Carpenter & Toftness, 2017; Grimaldi & Karpicke, 2012; James & Storm, 2019; Little & Bjork, 2016; Richland et al., 2009). That is, answering questions before reading or listening to a lecture presenting the to-be-learned material can improve the learning of that material when it is later read or presented in a lecture, especially the learning of the pretested information. Previous research has often found larger advantages of post-testing over pretesting (Geller et al., 2017; McDaniel et al., 2011), but sometimes the advantage of pretests match that of posttests (Pan & Sana, 2021). In this Special Issue, James and Storm (2025) investigated the relative benefits of pretests versus posttests using True/False tests and found that both types of tests led to a similar number of correct responses on the final test. For intrusions, however, True/False pretests led to fewer intrusions than did True/False posttests, even when significant feedback was provided on those posttestssuggesting that when True/False questions are used, pretesting may have advantages over posttesting.

Although testing clearly confers direct benefits to learning, testing can also provide indirect effects. Roediger et al. (2011b) have argued that testing can induce learning in a variety of ways, including providing learners with ideas about what they do and do not know and thus helping them to focus future study on information that is less well known, a point to which we will return in the section on Metacognition. Another indirect effect of testing is that testing some content can increase learning on futurto-be-learned content, the so-called forward testing effect (Pastötter & Bäuml, 2014; Yang & Shanks, 2018; Yang et al., 2018; see also early evidence in Thune, 1950). In this Special Issue, Assadipour et al. (2025) examined the forward testing effect using lecture videos. They compared a condition in which participants retrieved information about previously learned content to a control condition, as well as two other conditions that involved retrieval (i.e., semantic generation and autobiographical retrieval). Then, after watching a 15-min lecture video, participants took a test (or did one of the other retrieval activities), and then watched another 15 min lecture that was followed by another test. Participants’ performance on the most recently learned content (i.e., information about the second video) was facilitated by the retrieval of the earlier content as compared to the control condition; furthermore, semantic generation and autobiographical retrieval following the first video did not facilitate learning of the second video. Thus, it seems that regular test-taking of information that was learned will help learners to retain that information and will also help learners to retain new information that is subsequently presented.

In summary, testing is a powerful tool for learning because it can be used in a wide variety of ways and helps learning both directly and indirectly. Directly, recalling information makes that information more recallable in the future. Indirectly, retrieving information can have other advantages, including changing the effectiveness of future study, a point to which we return in the Metacognitive section below.

### 2.2. Study Schedules Influence Learning: Spacing and Interleaving Effects

Another desirable difficulty involves spacing out rather than massing study, the so-called spacing effect (Cepeda et al., 2006, 2008). Like the testing effect, the spacing effect dates back to early empirical records (Ebbinghaus, 1885/1913) and is also robust across a variety of materials, contexts, and types of learners (Carpenter et al., 2012, 2022; Dempster, 1989; Dunlosky et al., 2013; Fishman et al., 1967; Mawson & Kang, 2025; Rea & Modigliani, 1985; Seabrook et al., 2005; Sobel et al., 2011; Toppino & DeMesquita, 1984; Vlach et al., 2008). Several explanations have been proposed for the benefits of spacing. One prominent idea is that spacing introduces forgetting that, in turn, facilitates future learning (R. A. Bjork & Bjork, 1992). Spacing also increases the potential for encoding variability (R. A. Bjork & Allen, 1970), offering the opportunity to create multiple retrieval routes for later recall, a point to which we will return later.

Closely related to the spacing effect is the interleaving effect in which during category learning, for example, members of the different to-be-learned categories (e.g., Categories A, B, and C) are not presented in the more typical blocked manner (i.e., all and only examples of Category A, then all and only examples of Category B, then all and only those of Category C), but rather in an interleaved manner (e.g., one example from Category A with its label; then one from Category C, with its label; then one from Category B, with its label, and so forth until all the examples from each category have been shown). It was largely assumed that blocked presentation of exemplars would be more effective for the learning of each category than an interleaved presentation would be. Kornell and Bjork (2008a) examined this assumption for the learning of the painting styles of different artists and, surprisingly, found that interleaving the presentations of examples of the different artists’ paintings led to better learning of each individual artist’s style than did blocking the examples of each individual artist’s paintings. Interleaving benefits have been shown with a variety of materials (Brunmair & Richter, 2019; Firth et al., 2021; Pan, 2015; Rohrer, 2012; Rohrer & Taylor, 2007). Given this surprising result, many other studies have attempted to determine more specifically why interleaved category learning would be superior to blocked category learning. For example, research by Birnbaum et al. (2013) has shown that the observed interleaving benefit could not be attributed to spacing alone. A prominent idea for why interleaving is beneficial is that it allows learners to focus on differences between items from different categories (i.e., discriminative contrast e.g., Birnbaum et al., 2013; Kang & Pashler, 2012). Other possible (not mutually exclusive) reasons include the idea that interleaving increases attention (Wahlheim et al., 2011) or decreases mind-wandering (Metcalfe & Xu, 2016). Less robust than testing or spacing, the benefit of interleaving seems to depend more on contextual factors than does testing or spacing. For example, the strength of the effect is dependent on factors like the similarity of the materials (Brunmair & Richter, 2019), whether the items follow an articulable rule (Noh et al., 2016), whether participants can take notes and/or use them on the test (Little et al., 2026), and even individual differences in propensity to memorize or to find rules (Little et al., 2025; Little & Nepangue, 2025). One interpretation for why interleaving is sometimes worse for learning than blocking is that sometimes interleaving introduces too much difficulty to be desirable.

In summary, spacing out study is often potent for learning. Interleaving–a variation on spacing–can also be beneficial, but its benefits depend on more factors. Consequently, research continues to examine these circumstances so that learners and educators can effectively implement these strategies.

### 2.3. Metacognition: What Learners Know About Their Learning and How It Affects Their Study Choices

In addition to examining how to optimize learning, researchers have also been interested in what learners think about the effectiveness of their learning—that is, their metacognitions about learning. Research on metacognition has practical implications because metacognitive monitoring (i.e., what learners think about their knowledge) often impacts study decisions in the future (e.g., Koriat & Goldsmith, 1996; Little & McDaniel, 2015b; T. O. Nelson, 1996; T. O. Nelson & Leonesio, 1988; T. O. Nelson & Narens, 1990; Son & Schwartz, 2002). If, for example, learners think that they have already learned something well, they may spend less time studying it in the future; or, if they think that they have not yet learned something well, they may spend more time studying it in the future (e.g., T. O. Nelson & Leonesio, 1988). This seemingly obvious relationship, however, can sometimes be complicated by other factors, including, for example, what learners think they can reasonably learn in the available restudy session. When learners have limited time, they may focus their attention most on items of medium difficulty (region of proximal learning; Kornell & Metcalfe, 2006; Metcalfe, 2002; for a review of the relationship between monitoring and control, see Son & Schwartz, 2002).

In one study examining metacognitive monitoring and restudy decisions following practice testing or restudy of passages, Little and McDaniel (2015b) had participants read text passages about regions of the world (i.e., six regions, each divided into sections of Geography, Climate, and People, for a total of 18 sections; eight sentences/facts in each section). Then, the initial reading of the passages was followed by either a retrieval (practice testing) period in which participants retrieved all of the content they could remember or a restudy period. Participants then estimated the number of facts they would be able to recall for each section of text (e.g., Norway: Geography). Then, they were able to re-read a subset of the passages at their own pace, with the timing of each sentence measured. Finally, they took a free-recall test on information from all of the sections. In terms of metacognition, first, participants had more accurate metacognitive estimates in the retrieval condition than in the restudy condition. Second, when they had the chance to re-read the sentences from half of the sections, they spent more time rereading information from the sections for which they had given the lowest estimates of future recall than the sections they had given the highest estimates of future recall. Critically, their rereading strategies–following from their metacognitive estimates–led to larger gains from rereading in the retrieval condition than in the restudy condition. Thus, practice testing helped learning both directly and indirectly. That is, recalling information during the practice test increased retention, but it also changed how learners allocated their study time during the phase in which they re-read the sentences, thus increasing retention for that initially tested content even more. Interestingly, participants had more accurate metacognitive judgments in the retrieval condition than in the restudy condition; most likely because they based their metacognitive judgments on their estimated success of retrieval during the first test, which was a concrete indicator. In the restudy condition—in which they made judgments based on less concrete information (e.g., fluency)—they greatly overestimated how much they would remember and were less well calibrated in terms of what information they knew well and what information they did not.

As indicated by the above pattern of results, while some aspects of metacognition can be accurate, other aspects can be inaccurate. Learners can have higher confidence than warranted following some study activities, as compared to other study activities that are actually more effective. In Kornell and Bjork’s (2008a) research examining the efficacy of interleaved learning, for example, the majority of participants believed that they had learned an artist’s style better when paintings by that artist had been blocked during study versus when they had been interleaved with those of other artists, even though the majority of participants had actually learned better in the interleaved condition. In Roediger and Karpicke’s (2006) research examining the retrieval practice effect, participants who received relatively more study time during the learning phase predicted better later recall of the text passages than those who had received less study time but more testing time during the learning phase. The results, however, showed that those receiving relatively more testing time during the learning phase outperformed those receiving relatively more study time during the learning phase.

In addition to examining metacognition and performance within a single study, some researchers have examined metacognition more generally through survey research. In these cases, researchers can often obtain more information about learners’ beliefs. Several such studies are included in this Special Issue. For example, Hartwig and Rohrer (2025) examined learners’ beliefs about learning efficacy in spaced versus massed and interleaved versus blocked learning. They found that although spaced practice was judged as more enjoyable, nearly half of the participants failed to appreciate its effectiveness for learning. Appreciation of interleaving over blocking was worse: participants neither liked it nor thought that it was effective.

Most of the research on metacognition has examined students’ perceptions of their learning. Witherby et al. (2025a), however, also examined teachers’ and parents’ perceptions of learning strategies, finding that students, teachers, and parents held generally accurate knowledge about the benefits of testing and spacing, rating those strategies higher than most others (e.g., highlighting, rereading, massed study). This pattern suggests that knowledge about the science of learning is being disseminated. The question remains, however, as to whether even if students have some correct metacognitive knowledge, do they use it? Yuksel et al. (2025) examined the durability of students’ knowledge and their use of learning strategies following an intervention of teaching those strategies. Although learners tended to maintain their knowledge of effective strategies over time, their use of those effective strategies did not increase over time. Thus, although students can know about effective strategies, their implementation of them does not always occur. Implementation can be difficult because of time constraints and the planning needed.

One way to increase learning is to draw learners’ attention to important information. Learners may learn how to attend to information based on cues given by instructors such as by explicitly telling students that a given concept is important. Another way to draw attention to important to-be-learned information is to give it higher point values on an assignment (e.g., Knowlton & Castel, 2022; Castel et al., 2007), which might have implications for how to designate point values on rubrics (Shumaker et al., 2025).

Taken together, findings on the testing effect, spacing effect, and interleaving suggest that the primary contribution of the desirable difficulty framework is not simply the identification of specific techniques, but a broader reconceptualization of learning as a process in which short-term performance and long-term retention are often misaligned. Learners’ feelings of learning in the short-term affect their beliefs about their learning (i.e., their metacognition), which often leads to errors in such beliefs when it comes to long-term learning. Advancing the field will require further exploration of when and for whom such difficulties are desirable and how to increase the accuracy of learners’ metacognition.

## 3. Promoting Transfer: Using Learning in New Contexts

Although memorization and retention of information is critical, learners need to be able to take what they have learned in one context and apply it to another. Successful transfer of knowledge is inconsistent (Thorndike, 1906) and remains one of the major challenges in education (Barnett & Ceci, 2002). To transfer knowledge successfully, one needs both the ability to reason about the relationships between examples and to know when to do it (i.e., relational reasoning; Richland & Simms, 2015; Richland & Zhao, 2026).

### 3.1. Near Versus Far Transfer

One of the reasons that the successful transfer of knowledge is inconsistent is that successful transfer often depends on the similarity between the context in which information was learned and the new context (Thorndike, 1906). The more similar the contexts, the more likely the transfer. For this reason (among others), psychologists distinguish between near and far transfer, with the idea that near transfer evidences retention whereas far transfer evidences conceptual understanding (Barnett & Ceci, 2002). Barnett and Ceci provided a taxonomy for distinguishing between relatively near and relatively far transfer, considering changes in knowledge domain, physical context, temporal context, functional context, social context, and modality. Far transfer is more challenging because it requires the learner to abstract underlying principles from one context and recognize their relevance to another, which lacks overlapping contextual features (Gick & Holyoak, 1980, 1983).

### 3.2. Promoting Far Transfer

In their well-known paper, Gick and Holyoak (1980) examined analogical transfer. They provided participants with a story about a general invading a fortress with troops of soldiers that had to–owing to mines–attack using multiple small forces coming from different directions (i.e., convergence strategy). Later, participants were asked to solve a problem involving the destruction of a tumor using a laser (i.e., Duncker’s radiation problem, Duncker, 1945), which could also be solved using a “convergence” strategy. Most participants failed to see the connection between the general story and the radiation problem because the deep similarity (i.e., convergence solution) was obscured by surface-level dissimilarity (i.e., military vs. medical problem). However, certain factors could be introduced to help participants solve the radiation problem. For example, exposure to multiple stories with the same strategy but different surface-level details promoted the learning of the “convergence” rule and its application (Gick & Holyoak, 1983; see also Catrambone & Holyoak, 1989). Transfer can be successful when learners understand the concept decontextualized from a specific example. Relatedly, children could transfer a rule (“hide using mimicry”) to a new context to the extent that they understood the causal reason for the principle rather than having just memorized it (A. L. Brown & Kane, 1988). In this Special Issue, Richland and Zhao (2026) detail the critical role of this type of reasoning for education, arguing that relational reasoning is core to innovation and creativity (see also Markman & Wood, 2009).

Expertise in a domain depends on the ability to transfer knowledge (Chi et al., 1981; Ericsson & Smith, 1991). Experts develop a deep understanding of their domains and can readily see the deep structure without getting distracted by the superficial details. For example, Chi et al. (1981) showed that experts (i.e., graduate students) in physics could readily see problems utilizing the same underlying principle as conceptually similar, even when they lacked superficial similarity, whereas novices (i.e., undergraduate students) failed to do so. Novices were misled by superficial similarities across problems utilizing different underlying principles.

One way to develop a deep understanding of a domain is to think about the content flexibly and integrate it with one’s other knowledge. Note-taking can be valuable for conceptual understanding, as long as that note-taking is generative and non-verbatim (c.f., Mueller & Oppenheimer, 2014). Generative note-taking changes how learners process information, pushing them to decide what is important, to organize their knowledge, and to integrate it with prior knowledge (e.g., Fiorella & Mayer, 2015; Peper & Mayer, 1978). Generative note-taking should be especially beneficial for far transfer. For example, in an experiment on note-taking, Peper and Mayer (1978) found that overall, note-takers did better on far transfer questions than did non-note-takers, although note-taking did not help near transfer. Note-taking advantages also depended on learners’ level of background knowledge, showing evidence for the idea that learning depends on an interaction of factors related to the task, materials, participants, and test type.

Although desirable difficulties like testing and spacing are often used to promote simple retention, those conditions can be used to promote transfer as well. For example, Butler (2010) showed that testing was beneficial not just for recall, but also for one’s later ability to answer inference questions that tested the concept in a different domain (see also Van Eersel et al., 2016). Aligned with some arguments we have made so far, Butler argued that testing is beneficial for transfer because repeated testing with different questions should promote encoding variability and multiply retrieval routes in memory (c.f., Bower, 1972; Estes, 1955; Goode et al., 2008). That is, multiple retrieval routes can improve the likelihood of retention but can also improve the likelihood of transfer. Chan et al. (2006) showed that answering some questions about a text passage can facilitate recall of other related information, as long as the practice question could conceivably prompt a learner to think about related content when answering that initial question (see also Chan, 2009, 2010). We (Little, 2018; Little & Bjork, 2015; Little et al., 2012, 2019) showed that using multiple-choice questions with competitive alternatives can increase later recall of information pertaining to those alternatives–even though those questions were not specifically asked on the earlier test–because trying to reject the incorrect alternatives would induce a learner to think about why they are wrong and in so doing, recall related content. In fact, Little et al. (2019) used a protocol that involved asking participants to report how they answered multiple-choice trivia questions and found that although participants sometimes relied on recognition or “just knew the answer,” they sometimes reported using processes of elimination, with those processes often leading to the explicit retrieval of information pertaining to the incorrect alternatives. The benefit of multiple-choice questions for retention of tested and related information has been shown in the classroom as well (E. L. Bjork et al., 2014).

Spaced learning also introduces the opportunity for the encoding variability and/or study-phase retrieval processes that could multiply retrieval routes (Appleton-Knapp et al., 2005; Johnston & Uhl, 1976; Maddox, 2016). First, encoding variability could induce learners to think about a to-be-learned item in multiple ways. Second, to the extent that a subsequent presentation reminds one of an earlier presentation, learners may retrieve information about that earlier presentation, promoting covert retrieval (c.f., study-phase retrieval; Thios & D’Agostino, 1976). Not mutually exclusive, both theories could help to explain why spacing is beneficial for learning. Similarly, in interleaving, one can consider an example from one category contrasted with an example from another in one instance and in contrast to an example from a third or fourth category in others. Each comparison may induce the learner to think about a particular category in a different way. For example, considering Kornell and Bjork’s (2008a) painting materials, following one painting by Lewis with a painting by Cross might lead the learner to think about brushstrokes; following a different painting by Lewis with a painting by Braque may lead the learner to think about color.

## 4. How Individual Differences Influence Learning

Individuals have their own abilities (e.g., working memory capacity, structure building), background knowledge, and other characteristics (e.g., personality, propensity to memorize or find rules) that can affect their learning, with those individual differences sometimes interacting with conditions of learning to influence learning outcomes.

### 4.1. How Working Memory Influences Learning

Working memory is closely linked to attentional control. Working memory capacity is a relatively stable cognitive trait (Oberauer, 2019; Shipstead et al., 2016) and is widely studied as an individual difference measure (Smith-Peirce & Butler, 2025). Although the benefits of testing and spacing tend to be robust across a variety of learners, sometimes individual differences in ability, such as working memory capacity, can affect the efficacy of these strategies (Sana & Fenesi, 2025). In this Special Issue, Sana and Fenesi (2025) reviewed literature assessing how working memory influences the efficacy of desirable difficulties. Although working memory capacity has been shown to modify the efficacy of retrieval practice in some studies (Agarwal et al., 2017; Tse & Pu, 2012), most studies do not find a relationship (Smith-Peirce & Butler, 2025). For spaced repetition, the higher the working memory capacity, the bigger the advantage of a difficult intervening task (Bui et al., 2013), with these results predicting better advantages of larger spacing for high-working-memory-span individuals than for low-working-memory span individuals. Delaney et al. (2017) did not find that working memory capacity influenced the advantage of spacing, however. Further research is needed on this issue. Sana and Fenesi (2025) argue that educators (perhaps using appropriate technology) could adapt sequences of presentations to individual learners.

### 4.2. How One’s Propensity to Memorize Versus Find a Rule Influences Learning

Another individual difference worth examining is the strategy that people use to learn (e.g., memorization vs. rule-finding). As mentioned, experts are more likely to focus on deep similarities and novices on surface-level similarities (Chi et al., 1981), but some research has aimed to uncover whether individuals may differ in their propensities to memorize or figure out rules (Little & McDaniel, 2015a, 2015c; McDaniel et al., 2014; Juslin et al., 2003). That is, although much research in the science of learning has aimed at investigating the conditions of learning that are best for all learners, it is important to realize that learners bring their own propensities to the task. For example, Little and McDaniel (2015a) showed that when given a category learning task amenable to both rule-finding and memorization, some learners try to find a rule, whereas others focus on memorizing, with these different strategies leading to different learning outcomes (i.e., superficial retention vs. far-transfer, respectively). In their task, learners studied letter-strings from different categories (e.g., animal—blasimark, plant—spaksvot). The rule for category membership was that animals ended with “k” and plants ended with “t.” Later, they were tasked with classifying new items that looked similar to an old item but different in the last letter (e.g., blasimart). Those self-reporting as memorizers tended to classify the items based on similarity (e.g., blasimart is an animal), whereas those who self-reported as rule-abstractors tended to classify based on the rule (e.g., blasimart is a plant). Differences in lab-based function-learning tasks (i.e., predicting outputs for given inputs) using analogous rule-learning/exemplar-learner distinctions have been shown to predict performance in educational contexts (Frey et al., 2017, 2020; McDaniel et al., 2022).

The distinction between memorization and rule-abstraction seems to align with the idea of superficial learning versus deep learning in the Student Approaches to Learning Framework (SAL; Biggs, 1993; Biggs et al., 2001; Entwistle & McCune, 2004). In this Special Issue, McDaniel et al. (2025) raised the question of whether individual differences in memorization versus rule-abstraction are predictive of self-reported student approach to learning scores (surface or deep, respectively). Participants completed the SAL self-report survey and a function-learning task. McDaniel et al. (2025) failed to find a relationship between SAL scores and function-learning performance. This may be because the two constructs lack overlap, but it could also be because student metacognition is inaccurate (Dunlosky & Metcalfe, 2008) and/or self-reports represent intended approaches, not what students are actually able to achieve (Frey et al., 2020). Future research is needed to understand the stability of individual differences in memorization versus rule-abstraction and what such differences have to do with related distinctions (e.g., superficial learning propensities vs. deep learning propensities).

We have already mentioned that which difficulties are desirable might depend on features of the learner. But might the desirableness of the difficulties also depend on what learners are trying to learn? Little et al. (2025) had learners complete a category learning task modeled on the one used by Little and McDaniel (2015a). Learners did response-feedback trials for four letter-strings from each of four categories, with category membership depending on the last letter of the item. Additionally, in this research, participants saw items from a given category either blocked or interleaved. Replicating Little and McDaniel (2015a), Little et al. showed that when learners reported trying to memorize, they tended to classify test items based on similarity to trained items, but when learners reported trying to find a rule, they tended to classify later items based on the rule. Furthermore, when learners were trying to memorize (either by their own initiative or because they were instructed to do so), interleaving was better than blocking for their later classification of items based on similarity to trained items, but when learners were trying to find a rule, blocking was better than interleaving for their later classification of items based on the rule. In this Special Issue, Little and Nepangue (2025) replicated this work using an instruction manipulation (i.e., participants were instructed to memorize or to find a rule) and showed the predicted effects (i.e., blocking better than interleaving when instructed to find a rule; interleaving better than blocking when instructed to memorize) and showed that these effects persist across a delay of 48 h. Note, however, that others have shown advantages of spacing even when learning conceptual information that involves rule-learning/transfer (e.g., McDaniel et al., 2013a, in a function-learning task), so a factor that may need to be considered is the difficulty in extrapolating the rule. One possibility is that if the rule is relatively easy to extrapolate, people will likely benefit from spacing, but if the rule is very difficult to extrapolate, people may benefit from blocking.

## 5. Practical Implications

The articles in this Special Issue address many issues at the intersection of cognitive psychology and educational practice. Although most early research was conducted in the laboratory, there have now been many attempts to examine the principles of effective learning in real-world contexts. In many cases, the benefits of spacing and testing have emerged even in these practical contexts. For example, the advantage of testing (e.g., via quizzes, clicker questions, etc.) tends to be shown in real classrooms (see review by Agarwal et al., 2021; meta-analysis in this Special Issue, Mawson & Kang, 2025; but see Moreira et al., 2019). Agarwal, McDaniel, Roediger, and colleagues have completed many studies on testing in authentic educational contexts (McDaniel et al., 2007a, 2007b, 2013b; McDaniel et al., 2011; Roediger et al., 2011a). Although classroom research is tremendously valuable, it is underrepresented in the literature because it often takes more resources and coordination to conduct than it takes to conduct lab-based research. Additionally, classroom research must often sacrifice control for increased ecological validity and this lessening of control can sometimes reduce or eliminate effects, serving as a disincentive for investigators to perform this type of research. However, the reduction of effects does not necessarily indicate that the principles discussed here do not have ecological validity. To illustrate, imagine a within-subjects study implemented in a course using authentic materials to examine the efficacy of practice testing as a tool for learning. In an actual course, students will often study on their own to maximize test performance because grades are extrinsically motivating. If the instructor implements practice testing for some information, students may find that they need to study that information less but may study the other non-tested information more before the test, thus raising that baseline (control) performance. Thus, although practice testing helped, the baseline performance also improved. Such extra study is much less likely to happen in a laboratory experiment.

There can also be other challenges to implementing desirable difficulties in real-world situations—either on the part of the instructor or the student (Hertel, 2020). Testing is probably the easiest high-impact practice to implement in educational contexts. Most obviously, testing can include quizzes and exams, but less obviously, testing can include clicker or polling questions (Deslauriers et al., 2011), think-pair-share activities (Kaddoura, 2013), or review games (Iwamoto et al., 2017). Students can also test themselves. The first author of the present paper implements the aforementioned three strategies and often encourages her students to leave class trying to recall at least one thing that they learned.

Spacing and interleaving are more challenging for teachers to implement because they involve teaching information in a non-linear manner or circling back to information. One relatively painless way for instructors to implement spacing is by making time for reviews at the end of each section and making exams and quizzes cumulative (or, if that is too aversive, including extra credit questions from topics earlier in the course). Instructors can also plan to revisit information in order to connect it with new information. The first author likes to do this in her cognitive psychology class, for example, by introducing the concepts multiple times throughout the semester while making different points about a given concept each time it is reviewed and reminding students that they learned something about this concept earlier in the course. Students can implement spacing through planning study sessions throughout the semester and using flashcards for spaced retrieval.

In order to induce transfer, teachers may want to give students many examples of a concept that differ in superficial features, drawing students’ attention to the deep structure or concept (Gick & Holyoak, 1983). For example, in their paper, Little and Nepangue (2025) suggest that when discussing context-dependent memory, one might highlight studies on environmental context (e.g., Godden & Baddeley, 1975), emotional context (e.g., Eich & Metcalfe, 1989), and the context of the processes involved during encoding and retrieval (Fisher & Craik, 1977), explicitly stating that although the studies may seem different on the surface (i.e., because they differ a lot in superficial features), they are all evidence for the same underlying process: encoding specificity. (Although most cognitive psychology instructors likely teach encoding specificity with a similar variety of examples, they may find that many of their students get stuck on the superficial details and fail to see the connection between the different studies unless they make the shared concept very explicit.) In their review, Richland and Zhao (2026) suggest, for example, that drawing attention to one-on-one relational correspondences within representations—sometimes with spatially aligned representations—may be crucial for ensuring that the learner recognizes the value of relational reasoning. Likewise, instructors may need to provide cues (e.g., “solve this problem the same way”). Richland and Zhao provide several additional suggestions for promoting relational reasoning, including building on prior knowledge and supporting novices in developing a relational mindset. To build a relational mindset, for example, an instructor could highlight the importance of relational information while explicitly downplaying the importance of object-based information.

As mentioned above, laboratory research is often conducted before classroom research because laboratory research is usually easier to conduct, and the stakes for participants are much lower in a lab-based context than in a classroom-based context. Some of the research introduced in this Special Issue has implications for learning in real-world contexts, but future research in real educational contexts should be conducted before making clear recommendations about real-world use. For example, Liu et al. (2025) showed that rewarding accurate metacognition increased performance as compared to rewarding performance (and to a control condition). A future study could examine whether rewarding accurate metacognition in the classroom by adding points on learning activities, quizzes, or exams for accurate metacognitive judgments could improve subsequent performance. Relatedly, justifying one’s answers in multiple-choice quizzes may improve performance (Clark et al., 2025), so researchers could examine whether answer justification on low-stakes quizzes improves performance on later exams. In short, how these research studies could be applied in classroom contexts is clear, but future research should examine whether the effects persist in those contexts.

## 6. Future Research Directions

As discussed above, there are many open areas for future research to investigate the application of research in cognitive psychology to educational contexts. A critical next step for the science of learning is to move from establishing the existence of robust effects toward establishing an integrative framework that more clearly specifies how these effects depend upon the nature of the material, task, learners, and perhaps other contextual features (c.f., Jenkins, 1979). Some papers in this Special Issue also expand upon the contextual features we should consider (e.g., demographic variables of participants, how technological advances affect how people learn). The directions highlighted below illustrate how interacting factors shape learning across domains, contexts, and in an educational environment in which technology is evolving very quickly.

Although we have highlighted testing, spacing, and interleaving as difficulties that are desirable for long-term retention and transfer, there are other difficulties that may be desirable for learning, many of which are discussed elsewhere (R. A. Bjork, 1994; R. A. Bjork & Bjork, 2011, 2020). Indeed, even more desirable difficulties are likely worthy of study. In this Special Issue, Li (2025) examined the extent to which language learners rely on captions while watching a video in a second language. Although learning language through movies and television should improve listening skills, relying on captions may lead learners to improve reading skills rather than listening skills (Vandergrift, 2004). Li found that less proficient learners tend to rely on captions more than do more proficient learners. It might be that less reliance on captions would be too difficult for a less proficient learner, but it might be the case that less reliance on captions is a desirable difficulty for all. This is an empirical question for future research.

Most cognitive research we have discussed here focuses on the individual, but some recent work has examined cognition in groups/collaborative cognition (Rajaram & Pereira-Pasarin, 2010; Rajaram et al., 2025). Although collaborative memory has been studied for some time, less well understood is collaborative metacognition. Metacognition during collaborative learning includes more than individual metacognition (Järvenoja et al., 2017; Järvelä et al., 2016). In this Special Issue, Wei et al. (2025) examined students’ metacognition about collaborative learning, and they showed that although students prefer group study, they perceive group study to be less effective than individual study. What is still unknown is how these metacognitive judgments relate to actual learning, and, thus remains a question for future research.

In this Special Issue, we have also highlighted the effect of learning strategies on learning outcomes. Other factors, such as motivation, may influence learning outcomes. For example, Smits et al. (2025) examined whether different break techniques influence motivation, productivity, and fatigue. Although they did not find any group differences between different break techniques on any of the variables they examined, further examination of how breaks influence learning seems an important area for future research.

Additionally, technology is constantly evolving in terms of how information is delivered and stored and is increasingly changing how learners engage during learning. As mentioned by Sana and Fenesi (2025), computerized programs or applications may be able to deliver optimum spacing/interleaving depending on aspects of the materials and the abilities of the learner. Also, the way in which information is presented and saved has changed over time. Laptops enable students to take notes more quickly. Being able to take notes more quickly, however, may have negative consequences for learning because it reduces the need to think deeply about what information one should write down (Mueller & Oppenheimer, 2014). For the last few decades, many professors have given lectures using slides, sometimes providing these slides to their students to prepare for the lecture or to study afterward. Students like having access to the slides, but research on the effectiveness of having the slides for learning is mixed (e.g., Grabe et al., 2005; León & García-Martínez, 2021; Weatherly et al., 2003). Of course, even if professors do not post their slides, students can quickly capture the content in class by taking pictures with their phones. It is important to understand how such practices might influence learning. Previous research has shown that photo-taking can impair memory of the photographed information compared to non-photographed information (Henkel, 2014; Lurie & Westerman, 2021; Niforatos et al., 2017; Soares & Storm, 2018, 2022), but in this Special Issue, we see that such impairment is not always the case (Trego et al., 2025). The rise of artificial intelligence and LLMs (large language models) in the last few years has changed more than just how information is delivered and stored—and it has the potential to drastically change how and what people are learning. A concern is that it will reduce the need to use effortful processing that supports long-term learning. Future research can examine how AI can be leveraged to improve rather than reduce learning.

Finally, more research is needed regarding how individual differences influence learning and how the effects discussed in this Introduction might be different for different groups of people. Recent critiques of reliance on WEIRD samples (i.e., people from Western, educated, industrialized, rich, democratic nations) should be considered (e.g., Rad et al., 2018), and it remains important to examine how these principles apply across more diverse populations. That said, even within the traditional groups studied in research, many potentially important individual differences have mostly been ignored within the cognitive literature (e.g., socio-economic status, race, first- vs. continuing-generation status of a college student). For example, a demographic shift in recent decades involves the presence of more first-generation college students (i.e., students who are the first in their family to attend college). It is important to include demographic differences, such as whether students are first- or continuing-generation students, in order to see how principles of the science of learning play out across these types of different populations (see, e.g., Arcos et al., 2025). Fortunately, there is some evidence that active learning (including some of the methods reviewed in the present paper) is even more beneficial for traditionally underrepresented students (Theobald et al., 2020), although much more research is needed on this issue.

## 7. Conclusions

The field of cognitive psychology has clear implications for educational practice. The field is vast, and this Special Issue and the present Introduction focused on only a few robust principles. To summarize, study strategies that feel easy for learners are not always desirable in terms of producing learning that is both durable and generalizable, and learners often fail to appreciate the desirability of these difficulties–at least when they are in the midst of learning (e.g., R. A. Bjork & Bjork, 2011). Research on such desirable difficulties (e.g., testing oneself, spacing out one’s study, and interleaving examples from different categories) has applications for how to construct more effective learning experiences. Considering desirable difficulties in light of other factors (i.e., of the individual, the materials, and the test) provides additional factors to consider when applying this knowledge, and considering these factors can increase our ability to predict learning outcomes. This Special Issue goes beyond these principles, however, by including 23 papers addressing a range of research at the intersection of cognitive psychology and education. The included papers provide more specification for the principles above, with replications of previous findings in new contexts, provision of novel findings, synthesis of previous literature (reviews/meta-analyses), and the introduction of new research questions. Advancing the field will require a more nuanced understanding of how principles described here interact with learner characteristics and educational contexts. As research continues to bridge cognitive psychology and education, the principles discussed here have the potential to shape how learning is designed and experienced across contexts, resulting in better teaching and more effective and efficient learning.

## Note

1 Note that there are many potential reasons for why testing, spacing, and interleaving improve learning. This paper is not meant to provide an exhaustive list of theories, but we will discuss some other theories later in this paper.
